# Materials discovery at BESSY

**DOI:** 10.1140/epjp/s13360-023-03957-8

**Published:** 2023-04-21

**Authors:** Olaf Schwarzkopf, Andreas Jankowiak, Antje Vollmer, for the BESSY II/BESSY III team

**Affiliations:** Helmholtz-Zentrum Berlin, Hahn-Meitner Platz 1, Berlin, 14109 Germany

## Abstract

The BESSY II synchrotron radiation source at Helmholtz-Zentrum Berlin (HZB) is an internationally leading facility playing to its strengths in the UV and soft X-ray regime, with the mission to enlight and enable materials discovery, develop solutions and answers to the societal challenges of this century, like Energy, Information and Health, and enable research and innovation along the entire value chain. To maintain BESSY II competitive while bridging to its successor source BESSY III, HZB is currently developing an ambitious strategic upgrade program of the facility which includes maintenance and modernization measures as well as the provision of new research opportunities with the focus on new *operando* capabilities for energy research and technology development. On the longer term, the 4th generation source BESSY III is needed to meet the requirements of the mission-oriented scientific focus fields Catalysis, Energy, Quantum and Information and Life Sciences as well as Metrology for Innovation.

## Introduction to the facility

**Fig. 1:**
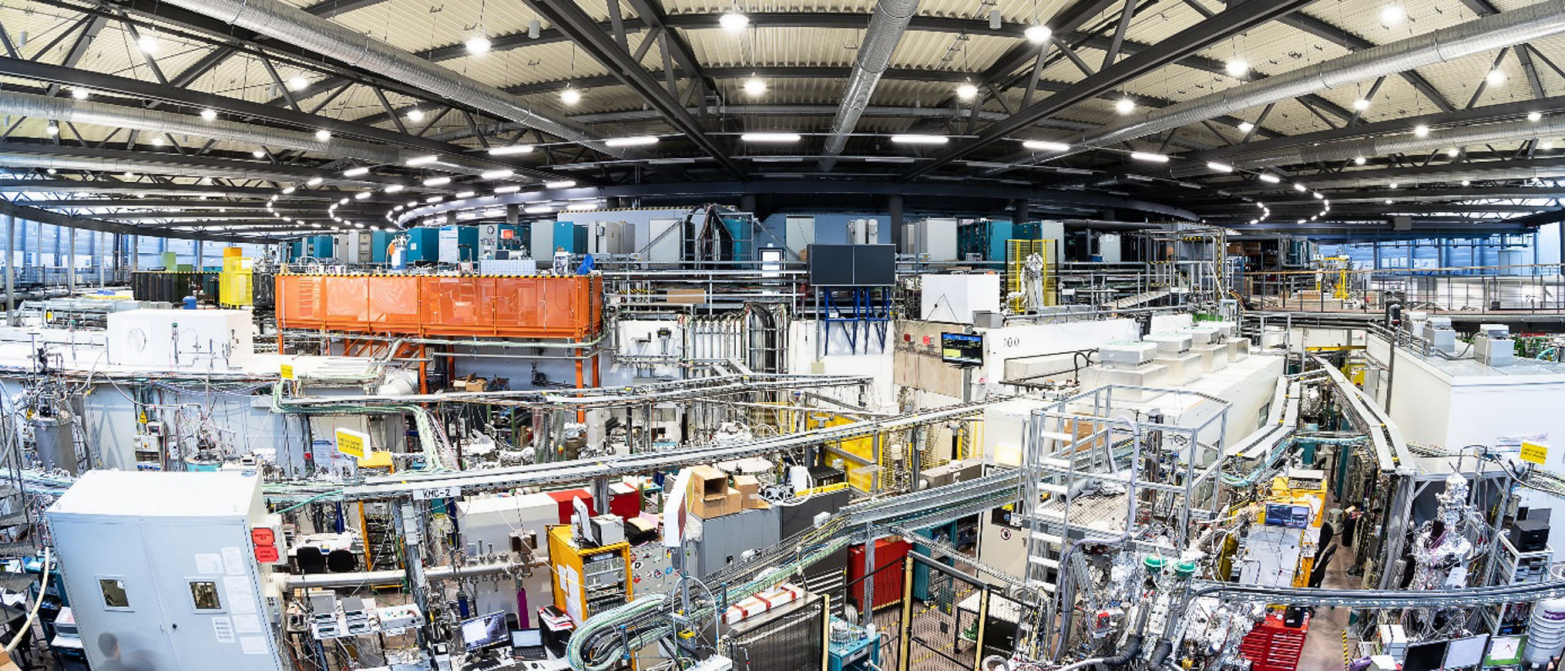
View of the BESSY II Experimental Hall

The BESSY II synchrotron radiation source at Helmholtz-Zentrum Berlin (HZB) is an internationally leading facility playing to its strengths in the UV and soft X-ray regime, with the mission to enlighten and enable materials discovery, develop solutions and answers to the societal challenges of this century, like Energy, Information and Health, and enable research and innovation along the entire value chain (Fig. 1).

The BESSY II source—a third-generation 1.7 GeV storage ring—features a sophisticated multi-purpose / multi-user fill pattern, providing high brightness operation together with customized bunch manipulation and separation techniques. This allows to serve both photon hungry and time-resolved experiments at the same time. This capability, together with purpose-made insertion devices, allows for exceptional energy, spatial, temporal, and polarization control of the photon beam. BESSY II is a radiation standard for X-rays and is used by Germany’s National Metrology Institute (PTB) for a wide range of metrology applications (see corresponding article in this volume). These activities as well as those of the German Federal Institute for Materials Research and Testing (BAM) at BESSY II also serve as bridge toward application-related research.

BESSY II serves an international user community spanning a great variety of disciplines. More than 1200 user proposals are submitted per year to the half-yearly calls, resulting in about 800 beamtime campaigns at the 38 beamlines currently in user operation, and approx. 500 verified publications per year. The involvement of HZB and partner research groups in beamline operation provides the basis for the strong scientific support for BESSY II users, ranging from the development of special accelerator operation schemes, dedicated instruments and sample environments to the evaluation and interpretation of the data. In addition, complementary laboratory infrastructure at HZB is available to BESSYII users for sample preparation and characterization [1]; prerequisite for its use is a granted beamtime application.

## BESSY II status and scientific highlights

With the implementation of Top-Up operation in the last decade, the beam current of BESSY II is stabilized to better than 0.5% and with injection efficiencies in user operation of 95% or above, a real “green” operation in terms of radiation losses could be established. With a continuous investment in replacing obsolete technology, the availability of BESSY II, with approx. 5000 h of scheduled user operation per year, reached a value of roughly 99% over the last 5 years. But not only the user performance is benefitting from these continuous investments, also energy efficiency improved by replacing the outdated klystron-based RF-system by a solid-state-amplifier based one with new HOM damped cavities.

BESSY II’s long-term strategic development is guided by the centre’s programmatic research, the involvement of HZB’s strategic partners, HZB’s scientific environment and the BESSY II user community as a whole. Within this framework, the areas of catalysis / electrochemistry and quantum and correlated materials have been identified as strategic priorities where investments will not only create new opportunities for materials discoveries, but also new bridges between basic research and industry. Moreover, the automatization and digitalization of the entire scientific workflow is crucial not only for lowering access barriers for non-synchrotron expert and industrial users. The Covid 19 pandemic has highlighted the importance of these methods and approaches in building institutional resilience. These findings are backed by our ISO 9001 certified quality management system for user beamtime projects, which ensure highest quality standards for the user service and provides guidance for the science- and demand-driven development of the facility.

**Fig. 2:**
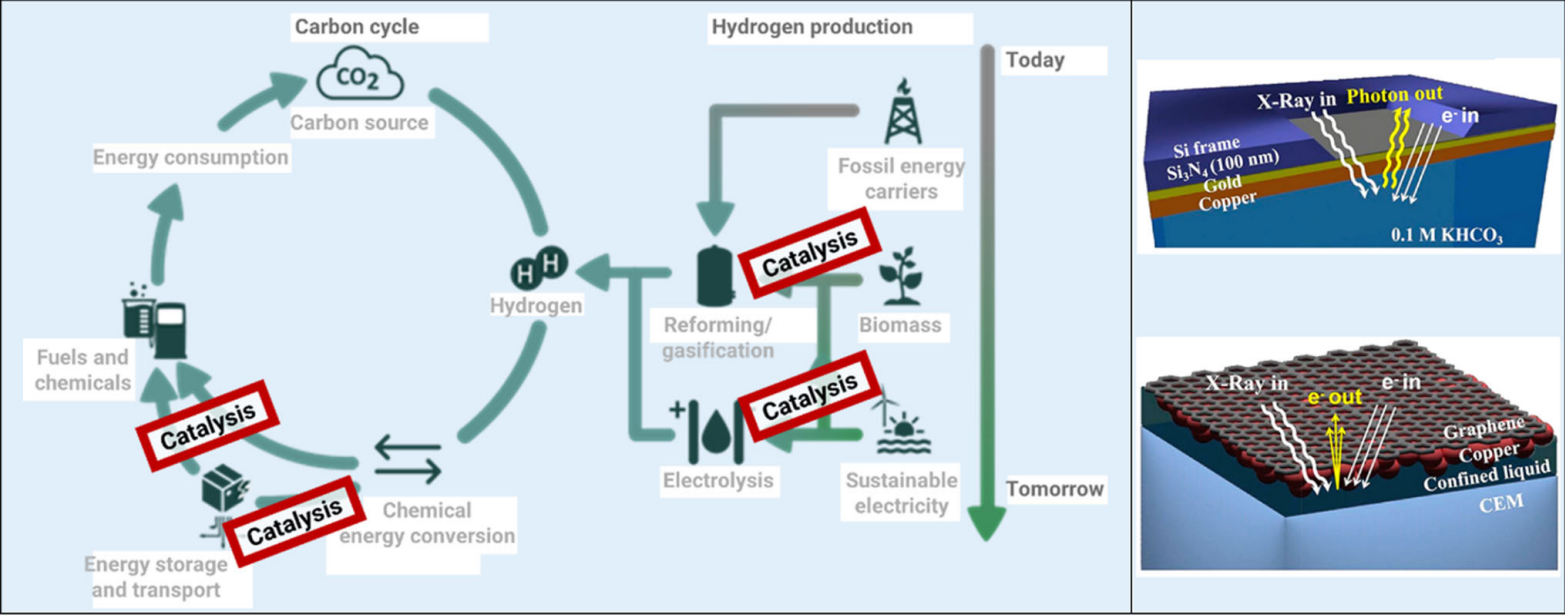
Relevance of Catalysis in a CO2 neutral energy system (left); cells developed for operando catalysis research, designed to be used for soft X-ray photoemission (top right) and fluorescence spectroscopy (bottom right) [2]

Experiments with soft-to-tender X-rays are an indispensable tool for materials research due to their ability to probe the electronic, chemical and spin structure by spectroscopic techniques or spectral imaging. For a wide range of applications, in particular for energy and quantum materials, the electronic structure is the key property that determines the functionality. Catalysis research (Fig. 2), e.g., within the recently started joint CatLab project of HZB and the Max Planck Society (MPG) is a prototypical example. CatLab has the objective to develop new types of catalysts based on thin films that efficiently convert chemical into electrical energy and vice versa. A knowledge-based approach is pursued which requires a complete analytical characterization of the catalyst under working conditions (*operando*). Here, the use of the possibilities of BESSY II for *operando* spectroscopy and microscopy on thin layers is a central element. For this purpose, existing instrumentation at the EMIL and ISISS laboratories is used and further developed, and new infrastructure is being built up as part of the Berlin Joint Lab for Electrochemical Interfaces BElChem [2] and the CATLab project [3]. Specialized equipment developed together with the Fritz Haber Institute of MPG has already enabled a number of scientific highlights in recent years, e.g., the identification of the active site in ethylene epoxidation over silver catalysts [4] or in the electrocatalytic oxidation of water over Iridium-based catalysts [5].

Expanding the CatLab activities and further bridging to industry, HZB as well as several academic and industrial partners from Germany and South Africa have started the project CARE-O-SENE that focuses on the development of Fischer–Tropsch catalysts for the production of green kerosene. Again, key elements for the project’s success are fast and reliable access to the analytic *operando* tools of BESSY II and an early involvement of key users and industrial partners, ensuring the transferability and scalability of the results into applications.

Experiments at BESSY II are also relevant for photovoltaics research, namely research on high-efficiency perovskite solar cells [6, 7]. For example, HZB’s tandem perovskite cells, which have set world efficiency records several times in the past years, were measured at BESSY II in collaboration with partners of the PTB [8]. PTB and HZB also actively collaborate on *operando* synchrotron measurements on batteries using the globally unique capabilities for materials metrology offered by PTB; one example being the quantitative analysis of sulfide movement between electrodes, which is identified as a key origin of capacity fading [9].

In the field of green IT, ARPES measurements are the basis for the highly productive research at BESSY II on the topic of spintronics, e.g., [10, 11], a field of materials science that aims at reducing the energy imprint of information technologies.

**Fig. 3:**
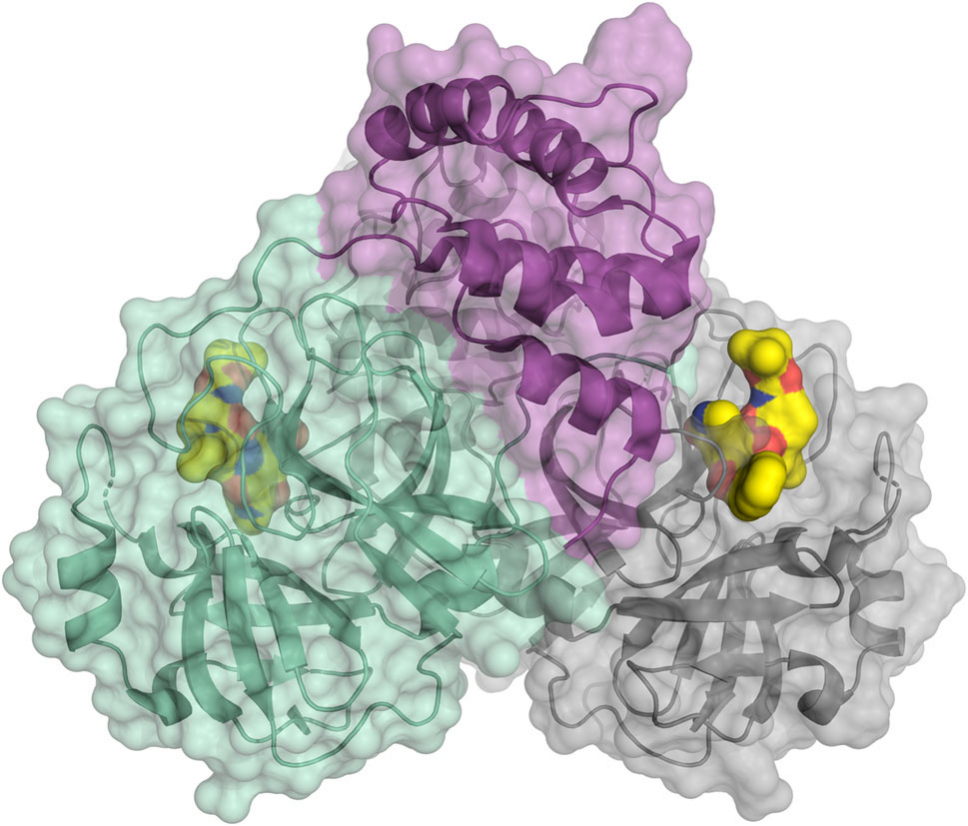
Schematic representation of the coronavirus protease, as determined from measurements at the Joint MX Lab at BESSY II [12]

Life Sciences at BESSY are driven by a strong and vibrant user community forming the Joint Berlin MX Laboratory which has allowed the decoding of the three-dimensional architecture of the main protease of the SARS-CoV-2 virus [12] (Fig. 3). The analysis of the 3D architecture of this protein allows the systematic development of drugs which inhibit the reproduction of the virus.

## BESSY II upgrade plans for the decade 2023–2033

BESSY II has now been in operation for more than 20 years and will remain in operation at least until 2035, overlapping with the friendly user operation of its successor source, BESSY III, which is expected to start full user operation in 2036. As laid down in the recently published preCDR [13], BESSY III, integrated in the materials science campus Berlin-Adlershof, will provide unique capabilities for the national and international materials and energy science community by synergetically combining:

- a state-of-the-art 4th generation green field synchrotron radiation source,
- its embedment in an integrated research campus at Berlin-Adlershof, and
- quantitative and metrological materials science capabilities leveraged by the world-leading expertise of Physikalisch-Technische Bundesanstalt (PTB).

Maintaining BESSY II competitive while bridging to BESSY III requires an ambitious strategic upgrade of the facility, which includes maintenance and modernization measures as well as the provision of new research opportunities with the guidelines:

- Expanding capabilities in the field of electrochemistry and catalysis with the ambition to provide worldwide leading, industry relevant experimental possibilities; here, CatLab serves as a blueprint for the further development,
- Maintaining leadership in quantum and correlated materials by upgrading instrumentation,
- Keeping competitiveness in Life Sciences by implementing fast and efficient high-throughput methods,
- Continuously developing and implementing novel accelerator operation and instrumentation concepts as well as access modes, and
- Maintaining the facility’s leadership as a key enabler of metrology with synchrotron radiation and strongly develop and roll out materials metrology capabilities together with PTB and BAM.

BESSY II, as any large-scale infrastructure, consumes a significant amount of electrical energy. Dwindling resources, together with rising energy costs and climate change are all requiring mid- to long-term strategies for reliable, affordable and carbon–neutral energy supplies. First steps toward a more energy-efficient operation of the facility are therefore essential parts of the upgrade of BESSY II.

**Fig. 4:**
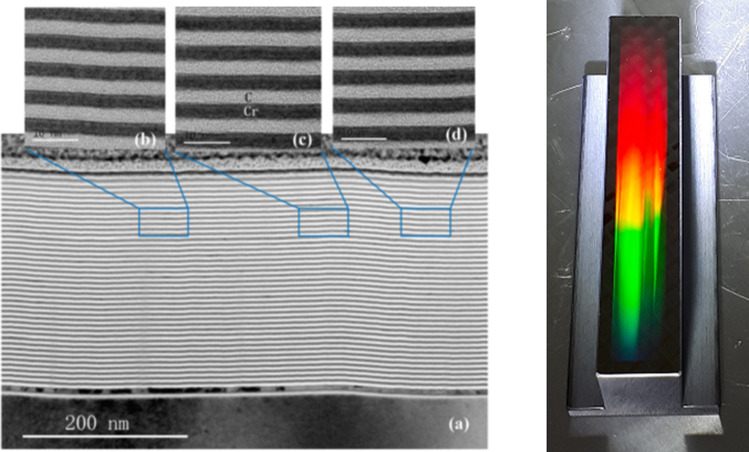
A cross section TEM-image of a whole layer stack of 40 bilayers deposited on a blazed grating structure [16] (left). 2000 l/mm ML-coated blaze grating of the U41-TXM-beamline at BESSY II (right)

The upgrade program will not only enable continued outstanding scientific work and innovation at BESSY II, but also initiate developments that are of great importance for the design of BESSY III. It will therefore also serve as a scientific and methodological bridge between BESSY II and its planned successor source BESSY III. Examples for these methodological developments are:

- 3rd harmonic normal conducting, HOM damped, active cavities are developed in a collaboration between the HZB, DESY and ALBA, with the final goal of installing it at the BESSY II ring to be tested with beam [14]
- In response to scientific needs to cover, within one beamline, the soft-to-tender X-ray range, a new monochromator design is developed utilizing multi-layer optics [15, 16]. By adding a multi-layer coating in addition to the standard gold coating for both the pre-mirror as well as the blazed grating (Fig. 4), the corresponding photon flux in the tender range is increased—at the prize of losing highest resolution—by more than two orders of magnitude.
- *In situ* and *operando* sample environments for electrochemical and catalytic experiments have been established, for example standardized battery cells and new interchangeable sample environment modules for standardized soft X-ray end-stations. New, advanced *operando* sample environment and infrastructure is being built, e.g., to combine high pressure catalysis with soft X-ray absorption spectroscopy based on micro-electro-mechanical systems (MEMS)- reactor technology [17]

## Perspective of next scientific challenges to be addressed at BESSY III

The structural complexity of modern materials, often containing inherent or tailored nanoscale structural elements, as well as the need to expand the capabilities of *operando* and *in situ studies* often requires experimental possibilities beyond those of today’s SR sources. In collaboration with its users and partners, HZB has therefore developed a concept for a 4th Generation successor source, BESSY III, that matches the requirements of the mission-oriented scientific focus fields Catalysis, Energy, Quantum and Information and Life Sciences as well as Metrology for Innovation.

In **Catalysis**, BESSY III will provide dedicated instrumentation to study the chemical states in particular of (sub-)surface species which are involved in catalytic reactions, and thereby yield an atomistic understanding of the reactions mechanisms which is only accessible in *operando* investigations. This understanding will form the basis for developing and validating novel concepts and materials for catalytic applications. Collaborations with industrial partners at an early stage, as established in the CatLab project for example, will ensure a focus on the large-scale practicability of these concepts. In addition, CatLab also serves as a blueprint for novel, more flexible “challenge-driven” access modes which will be implemented at BESSY III.

For **Energy research**, BESSY III will combine dedicated, highly automated measurement and sample environment capabilities with advanced beam properties to enable *operando* studies of processes in energy devices on a wide variety of length and time scales, embedded in associated facilities for synthesis and characterization. This will permit fast yet knowledge-based device structure optimization, e.g., by identifying essential loss mechanisms, scanning large parameter spaces for the optimal material composition, and evaluating degradation mechanisms. Thus, novel approaches for energy conversion or storage devices can be efficiently probed for their potential to be developed into applications.

By providing spectroscopic and imaging techniques with high spatial and spectral resolution, BESSY III will identify promising candidate materials for novel computing applications based on quantum effects. The high spectral brightness of the source will permit to develop such investigations toward *operando* studies of prototype devices, thereby taking a further step toward **Quantum and Information Technologies** based on the implementation of these materials and phenomena.

High-resolution X-ray imaging and high-throughput macromolecular crystallography at BESSY III will constitute essential elements of a versatile toolbox for **Life Sciences**. Using this toolbox, in combination with advanced modeling approaches, will deepen our understanding of biological processes, in particular molecular processes involved in diseases, such that faster and more targeted drug search becomes feasible. Based on a thorough general understanding of such processes and the establishment of substance libraries, this will also enable not only a very quick response to the emergence of new pathogens but also the development of drug therapies to treat major diseases such as cancer and dementia.

PTB will be able to extend its world-leading position in **metrology** with SR radiation by using BESSY III for (soft) X-rays and PTB's own metrology light source (MLS) or its successor (MLS II) for lower photon energies, in particular also as primary source standards. The availability of these experimental capabilities is urgently needed in many fields of high relevance for the future economy of Europe, including the semiconductor industry and sustainable energy technologies. Making use of the advanced source properties, quantitative measurements offered to industry and academia will be extended toward investigating complex structures on the nanoscale as well as materials and devices under real operating conditions. Accurate experiments generating quantitative data, offered by PTB, BAM, and HZB, will unleash the full potential of multimodal approaches at BESSY III.

The BESSY III main design criteria to fully respond to the needs of the scientific focus fields are (i) diffraction limited radiation at a photon energy of 1 keV and (ii) highest spectral brightness at 1 keV on the first harmonic of the undulators in combination with total spectral coverage in one beamline from 100 eV to some keV into the tender region. The lattice design (2.5 GeV ring energy, horizontal emittance 100 pmrad) is based on the MBA design and innovative magnet technology including permanent magnets for dipoles and hybrid magnets for a large fraction of the multipole magnets, thus gaining in energy efficiency of the storage ring. In order to make best use of the low emittance property of the electron beam, robust but innovative undulator concepts like double period devices (DOPU) as well as cryogenic (CPMU) and elliptical (IVUE) in-vacuum undulators will shape the photon beam, allowing for highest possible spectral brightness (i.e., an increase by 1–2 orders of magnitude compared to BESSY II) and coherent flux. Tailored soft-to-tender beamlines based on innovative monochromators and optics enable the user to benefit from a next generation electron storage ring. Supported by full automation of controls, diagnostics, AI and modern data management, the source characteristics will boost *operando* techniques and high-throughput materials discovery.

**Fig. 5:**
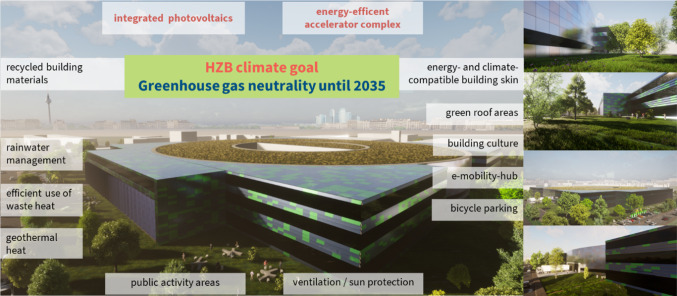
BESSY III—a Sustainable Research Infrastructure

With BESSY III, HZB is aiming at the highest possible standards of sustainable building as defined by the German Sustainable Building Council (DGNB). The DGNB evaluation scheme considers the overall performance of a building based on the criteria environmental quality, economic quality, sociocultural and functional quality, technical quality, process quality, and site quality. Bearing in mind that the reduction in energy consumption is limited for a facility like BESSY III, key aspects will be greenhouse gas-neutrality and recyclability of building materials as well as the energy-efficient and sustainable operation of the accelerator-beamline-laboratory complex, including the technical infrastructures for ventilation, cooling and heating (Fig. 5). The use of permanent magnets in the accelerator complex, an activated building skin and the usage of geothermal sources have the potential for substantially lowering the electricity consumption of the facility.

## Data Availability

Except for the Graphics on the left of Fig. 2, in Figs. 3 and 5, this review article refers exclusively to the results of the publications listed below and their references. New data are not associated in the manuscript. This manuscript has no associated data or the data will not be deposited. [Authors’ comment: There are no associated data available.]
